# Optical stimulation of neural tissue

**DOI:** 10.1049/htl.2019.0114

**Published:** 2020-06-25

**Authors:** Rachael Theresa Richardson, Michael R. Ibbotson, Alexander C. Thompson, Andrew K. Wise, James B. Fallon

**Affiliations:** 1 Bionics Institute, Melbourne 3002, Australia; 2 University of Melbourne, Medical Bionics Department, Melbourne, 3002, Australia; 3 University of Melbourne, Department of Surgery (Otolaryngology), Melbourne, 3002, Australia; 4 National Vision Research Institute, Australian College of Optometry, and Department of Optometry and Vision Science, University of Melbourne, Melbourne, Australia

**Keywords:** brain, ear, genetics, biological tissues, diseases, neurophysiology, bioelectric potentials, eye, gene therapy, reviews, neural tissue, electrical stimulation, deep brain stimulation, retinal stimulation, neural activation, low quality therapeutic outcome, direct optical stimulation, infrared light, light sensitive ion channel, infrared neural stimulation, cochlear implant, pacemakers, electroceutical treatment, disease, visible light, optogenetics, review

## Abstract

Electrical stimulation has been used for decades in devices such as pacemakers, cochlear implants and more recently for deep brain and retinal stimulation and electroceutical treatment of disease. However, current spread from the electrodes limits the precision of neural activation, leading to a low quality therapeutic outcome or undesired side-effects. Alternative methods of neural stimulation such as optical stimulation offer the potential to deliver higher spatial resolution of neural activation. Direct optical stimulation is possible with infrared light, while visible light can be used to activate neurons if the neural tissue is genetically modified with a light sensitive ion channel. Experimentally, both methods have resulted in highly precise stimulation with little spread of activation at least in the cochlea, each with advantages and disadvantages. Infrared neural stimulation does not require modification of the neural tissue, but has very high power requirements. Optogenetics can achieve precision of activation with lower power, but only in conjunction with targeted insertion of a light sensitive ion channel into the nervous system via gene therapy. This review will examine the advantages and limitations of optical stimulation of neural tissue, using the cochlea as an exemplary model and recent developments for retinal and deep brain stimulation.

## 1 Introduction

Electrical stimulation can be used to manage neurological conditions such as heart arrhythmias, hearing loss and movement disorders, and are in development for many other conditions, including blindness. Interfacing with the neural tissue is typically achieved via an array of stimulating electrodes positioned close to the nerve, externally controlled by a microprocessor to restore function that has been lost to disease. A large percentage of neural prosthesis recipients receive significant benefit from the use of their devices; however, electrical stimulation has some limitations that restrict the potential of these devices. In the case of cochlear implants, despite many patients achieving open-set speech discrimination in quite conditions, improvements in performance have plateaued in the last decades [1], and many recipients struggle, particularly in difficult listening conditions. Lack of specificity, off-target effects and current spread are the root causes of many issues of electrical stimulation. Replacing the electrical stimulus with an optical-based stimulus has the potential to address some of these issues. This review will explore the advantages and disadvantages of optical stimulation of neural tissue.

## 2 Electrical neural stimulation

Neural prostheses are implantable devices designed to apply electrical pulses to the central or peripheral nervous system for a therapeutic application. Arguably the most successful neural prosthesis is the cochlear implant which has restored hearing to more than half a million people with severe to profound hearing loss. Damaged or lost sensory hair cells in the cochlea are bypassed by the device which directly stimulates the auditory neurons with electrical pulses. Cochlear implant recipients can correctly recognise, on average, 82% of sentences in quiet listening conditions [2]. However, despite significant development and improvements to the electrodes and stimulation strategies over time, it remains difficult for recipients to understand speech in challenging listening conditions (e.g. background noise) and less than half of study subjects were able to recognise even the most well-known melodies or correctly identify musical instruments [3].

To represent complex sounds with a cochlear implant, very fine control over the site and timing of neural activation is required. Cochlear implants take advantage of the tonotopic organisation of the cochlea by processing sound into frequency bands and applying electrical stimulation to one of the 12–22 electrodes along the array. In theory, electrical stimulation of a single electrode should excite a well-defined cochlear region providing an independent channel of information. In practice, however, neural excitation is spatially very broad due to the conductive nature of cochlear fluids [4–7]. As a consequence, any single neuron can be activated by multiple electrodes, and thus by a wide range of acoustic frequencies, distorting the signal. Implant recipients therefore perceive low resolution spectral information [2]. Strategies that focus electrical current, such as multipolar electrical stimulation, can improve spatial resolution but at the expense of higher power requirements [7, 8] leading researchers to seek alternative forms of neural stimulation such as optical stimulation.

## 3 Optical neural stimulation

**Fig. 1:**
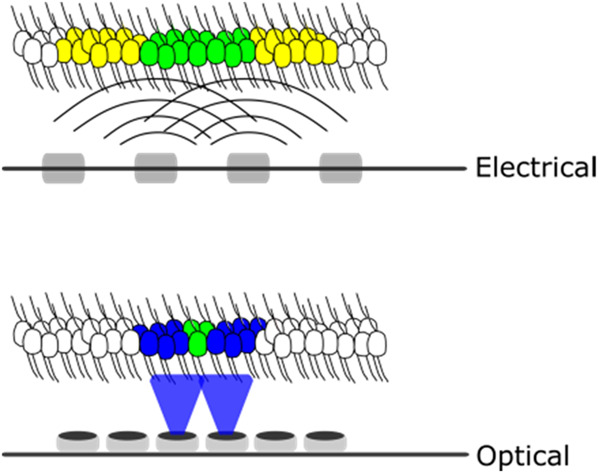
Neighbouring stimulating electrodes activate broad areas of neural tissue (yellow) with considerable overlap of activation (green). Focused optical stimulation activates a more discrete neural population (purple) whilst minimising overlap (green)

Optical stimulation has the potential to provide stimuli that are significantly more focused than electrical stimulation which may enable activation of more discrete, independent populations (Fig. 1).

There are two main forms of optical neural stimulation, infrared neural stimulation (INS) in which neurons can be directly stimulated without modification and optogenetic methods which first require genetic modification of neurons with a light-sensitive molecule to enable optical responsiveness.

### 3.1 Infrared neural stimulation

INS is a direct method of optical stimulation that has been shown to elicit action potentials in neural tissue [9]. The energy of infrared (0.95 nm–2.5 μm) and near-infrared light (750–950 nm) is hypothesised to be converted to heat causing activation of heat-responsive TRPV4 channels [10] and a change in membrane dimensions that affects membrane capacitance [11, 12], although the exact mechanism it yet to be determined. As INS relies on water absorption of light, the penetration depth can be tuned by selecting light wavelengths that have stronger or weaker absorption. Measurements of auditory brainstem responses show that cochlear INS activates auditory neurons in guinea pigs and gerbils [13–16]. Compared to electrical stimulation, 1.86 μm INS was shown to activate a more discrete area of the cochlea, with the spread of excitation being similar to acoustic stimuli [17]. In addition to the cochlea, INS has been used to stimulate the visual cortex [18], the sciatic nerve and other peripheral nerves [19].

In the cochlea there has been controversy about whether INS activates the neurons directly or whether the response is the result of a photoacoustic effect, whereby a pressure wave is generated from the optical pulse as it heats the fluid in the cochlea and activates the auditory pathway via residual hair cells. On the one hand, the INS response is compromised in profoundly deaf cochleae in which all hair cells have been ablated by chemical deafening [20] and the time dependent characteristics of the compound action potential response are indicative of photoacoustic activation [21]. On the other hand, INS appears to be a significant contributor to the neural response in the cochlea of *VGlut3* knockout mice in which hair cells are present but are lacking neurotransmitters, suggesting direct activation of auditory neurons [22]. These studies suggest that there may be multiple mechanisms responsible for the resulting response to INS.

**Fig. 2:**
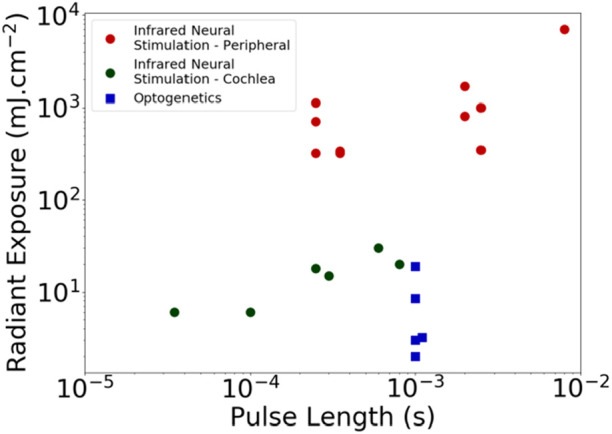
Radiant exposure of different optical stimulation techniques for different pulse lengths [9, 14, 19, 23, 26–38]. Thresholds for optogenetics-based stimulation in the auditory system vary depending on opsin used and transfection levels

There are two main restrictions of direct INS. First, very high energy pulses (17.2 ± 13.9 μJ/pulse) are required for neural stimulation in the cochlea, depending on the size of the optical fiber diameter and pulse length used [23, 24] and up to 1 mJ/pulse for some peripheral nerves [25]. Fig. 2 shows the radiant exposure (pulse energy per area) for INS in peripheral targets, INS in the cochlea and optogenetics-facilitated optical stimulation in the cochlea and brainstem.

For cochlear implant applications, this is at least two orders of magnitude more than for electrical pulses (0.2 μJ/pulse) [39]. Second is the potential to thermally damage the tissue with net heating of tissue dependent on both the stimulation rate and radiant energy. INS, at least in short-term experiments in the guinea pig, appeared not to affect compound action potential amplitude or cell survival when stimulating at 250 Hz and <25 μJ/pulse [40], but the long-term effects have not been examined. Introducing an intracellular absorber into auditory neurons, in this case silica-coated gold nanorods that were modified in shape to absorb light in the near-infrared range, was shown in vitro to help localise the energy, thereby reducing overall tissue heating and lowering energy requirements [41]. The superior tissue penetration of near-infrared wavelengths should also lower the energy required for neural activation compared to INS [42]. Gold nanorods have low toxicity [43–45] and can be targeted to cells for specific localisation [46]. However, the requirement for gold nanorods negates the main benefit of INS which is the ability to generate an auditory percept from optical stimulation without any manipulation of the neurons.

INS has not gained as much momentum as optogenetically mediated methods of optical stimulation but remains a viable tool for focused stimulation of neural tissue without the need to genetically alter the target tissue and is still being investigated for applications such as cardiac pacing and hearing restoration.

### 3.2 Optogenetic neural stimulation

**Fig. 3:**
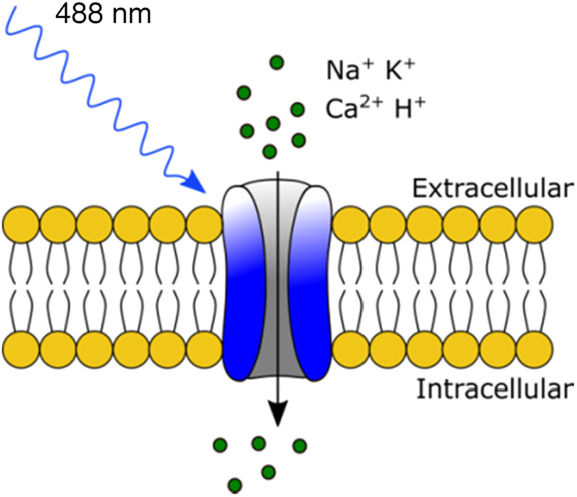
Optogenetic neural activation. A microbial light-sensitive ion channel such as channelrhodopsin-2 is genetically introduced into the membrane of neurons. Upon irradiation with light of a specific activation wavelength the ion channel opens and cations flow through the pore, resulting in depolarisation

Optogenetics is the combination of optics and genetics for the control of cells. Unlike direct INS, optogenetics first requires the genetic modification of neurons with light-sensitive ion channels called opsins to make them responsive to visible light [47]. Microbial type I opsins require covalent binding of a co-factor called all-*trans* retinal for photon absorption which triggers a rapid and reversible conformational change to allow the passage of monovalent and divalent cations through these membrane-spanning ion channels [48] (Fig. 3). All-*trans* retinal is found in mammalian tissues allowing type I opsins to be used as optogenetic tools for neural modulation. Eukaryotic type II opsins are G-protein coupled receptors which can modify intracellular processes with light, even in non-neural tissue, but are not the focus of this review.

Channelrhodopsin-1 and channelrhodopsin-2 (ChR2) were the first opsins to be isolated and characterised [49]. They originate from the green alga *Chlamydomonas reinhardtii* and ChR2 has gone on to be widely used in research. When expressed in mammalian cells (either by the generation of transgenic animals or through viral mediated gene transfer techniques), reliable control of neuronal spiking was achieved with pulses of blue light (peak 470 nm) without affecting neighbouring neurons that did not express the opsin [50]. However, ChR2 has low light sensitivity and relatively slow channel closing kinetics of nearly 10 ms, which limit its use in many clinical applications such as restoration of vision or hearing. Naturally occurring variants of opsins or directed mutagenesis have been found to favourably alter opsin properties such as channel closing kinetics, activation wavelengths, photosensitivity and ion selectivity, making optogenetics-based neural prostheses more feasible.

While activation of neurons in the visual system can be reliably evoked with 50 Hz stimulation rates [51], faster channel kinetics may be desirable in some systems such as the auditory system where modulations above 300 Hz are important for pitch perception [52, 53]. The CheTA variant of ChR2 was engineered for ultrafast neural stimulation by mutating residues in the retinal binding pocket, particularly the E123 T amino acid substitution, which decreased the conducting state from 9.2 to 4.4 ms [54]. Reliable spiking was observed with stimulation rates of up to 200 Hz without spike failures, improved from 20 to 40 Hz for ChR2. Unfortunately, faster kinetics often come at the expense of photosensitivity [55] but there are some exceptions. For example, CatCh was engineered via an L132C substitution that resulted in six-fold increased calcium permeability. Calcium not only induces faster response kinetics in the neuron via calcium-dependent potassium channels that help to hyperpolarise the cell, but CatCh is also 70 times more light-sensitive than ChR2 due to voltage-gated Na^+^ channel activation [56]. Another exception is Chronos, a naturally occurring channelrhodopsin that is excitable by green light (peak 530 nm). Chronos has high photosensitivity, a turn-on rate of 2.3 ms which is three times faster than ChR2 and a fast closing rate of 3.6 ms [57].

Blue and green light wavelengths are typically highly absorbed by blood which reduces tissue penetration and, therefore, effectiveness of optical stimulation in vivo. Chrimson is a naturally occurring variant that has a red light spectral peak at 590 nm [57]. Compared to other red-shifted variants such as ReaChR, which can be activated by both red and green light [58], Chrimson and its counterpart with improved rate-following ability, ChrimsonR, are more independent of the blue/green channels, potentially allowing two-colour control of different neural populations [57]. Another modification of ChR2, a single C128S mutation, extended the open state of the ion channel to 1.7 min following a single pulse of low intensity 470 nm blue light. Unlike other opsins, this so-called step-function (or bi-stable) variant can be inactivated by green light (560 nm peak) which means that the period of time that the channel stays open can be tightly controlled [59].

A significant advantage of optogenetics over electrical stimulation is the ability to directly inhibit neural activity with inhibitory opsins with clinical applications such as pain control [60] or seizure suppression [61]. Inhibitory opsins are light-gated chloride channels or outward proton pumps that hyperpolarise the neuron [62]. Examples include halorhodopsins isolated from halobacteria and archaerhodopsins that are activated by yellow or green-yellow light, respectively. Co-expression of excitatory and inhibitory opsins open up the possibility of bi-directional control of neurons with different wavelengths of light [63].

## 4 Applications for optogenetic neural stimulation

### 4.1 Optical cochlear implant

In the first demonstration of optogenetically mediated activation of the auditory pathway, it was found that activation thresholds were up to 75-fold lower in energy (2 μJ/pulse) compared to infrared cochlear neural stimulation (17.2 ± 13.9 μJ/pulse) [24, 64]. Comparing using radiant exposure rather than energy reduces the difference between the two techniques (Fig. 2). Importantly, the spread of activation in the brain was remarkably similar to an acoustic stimulus suggesting that an optically based cochlear implant can improve the precision of neural stimulation and hence the way sound is perceived [35, 64].

Initially, transgenic mice and rats with ChR2 expression in cochlear neurons were used, as well as embryonic viral mediated gene transfer that resulted in expression of the CatCh variant of ChR2 in ∼50% of neurons in the cochlear basal turn [64]. It was clear, however, that clinically translatable methods needed to be developed for the genetic modification of cochlear neurons. In 2018, postnatal mouse cochleae were directly injected with an adeno-associated virus (AAV) carrying the f-Chrimson variant, which has faster kinetics than ChR2 and is activated by red light. With up to 80% transduction of cochlear neurons, optical evoked auditory brainstem responses (oABRs) were recorded with 0.5–5 μJ of light even up to 9 months after gene therapy [65]. Demonstration of such long-term expression of opsins in neurons was a crucial step forward. Similar optogenetic neural activation was demonstrated following the introduction of Chronos via cochlear AAV injection in postnatal mice. Chronos allowed a much higher maximum stimulation rate, with neurons having a higher spiking probability during 0.5–1 kHz pulse trains, but required higher intensity light (7–14 μJ) and therefore more power [38].

Poor transduction of cochlear neurons in adult mouse models remained an issue, leading to the more extreme measure of directly injecting AAV viral vectors into the bony compartment housing the cochlear neurons in adult gerbils. Approximately 30% of cochlear neurons expressed CatCh via this method. Chronically implanted gerbils exhibited oABRs and behavioural responses to optical stimulation over weeks, even in animals with as little as 10% neural transfection. The average threshold was 4.6 mW for a 1 ms pulse (4.6 μJ) delivered at 10 Hz [35, 66]. There was, however, a loss of 25% of auditory neurons as a result of the injection technique. In another study addressing variability in ChR2 expression levels in individual cells, higher photosensitivity and probability of firing was found for cells with higher ChR2 expression, with overall influence on the oABR [67]. Safe and efficient delivery of opsins to spiral ganglion neurons remains to be tested in non-human primates.

Optogenetic neural stimulation of the auditory system has delivered improved precision of activation in the cochlea using lower energy than INS, but the overall power requirements still remains at least ten times higher than electrical stimulation alone, which is a big issue for battery-dependent devices. Additionally, successful implementation will require justification of the need to genetically modify the neurons. Significant benefits of improved precision of neural activation will need to be established to offset the additional gene therapy requirement. In particular, it is necessary to prove that the gene therapy procedure is safe and non-damaging to the delicate sensory cells of the cochlea, such as the residual hair cells and the auditory neurons. Currently, there is no indication that expression of ChR2 in neurons has any impact on their normal function or cell survival, including in non-human primates in which ChR2 was successfully delivered to the frontal cortex and activated with light over many months [68]. The use of viral vectors for human gene therapy is proving to be safe and effective in many tissues; however, current experimental gene therapy techniques in the cochlea can have a negative impact on hearing thresholds as cochlear gene therapy typically requires local injection into the cochlear fluids which can affect the survival of residual hair cells or their synaptic connections to auditory neurons [69, 70]. Furthermore, in non-mammalian rodents and small mammals it has proven to be more challenging to transfect auditory neurons with viral vectors in comparison to other cells of the cochlea such as hair cells [71]. The question of whether these issues translate to humans remains to be determined, taking into account that cochlear implant candidates typically do not have functioning hair cells and that cochlear implantation in itself can cause damage to residual cochlear structures. Clinical trials assessing the safety, tolerability and efficacy of viral gene therapy in the cochlea are underway for hair cell regeneration (NCT02132130) and electrically mediated gene transfer (ACTRN12618001556235). Research into gene therapy in the cochlea is intensifying as more and more studies are showing encouraging results relating to the repair of genetic defects in animal genetic models of hearing loss [69]. It will be critical that channelrhodopsin expression is long-term, preferably life-long in order for an optical device to be operational over the lifetime of the recipient. Gene expression via AAV has been shown to be long-term in human studies [72]. More specifically, ChR2 expression for as long as 64 weeks has been demonstrated in rat retinal ganglion cells following intra-vitreous adeno-associated viral delivery of the gene [73]. The combined outcomes of these studies will help establish the possibility of transfecting the auditory neurons safely and effectively for optical stimulation.

### 4.2 Optogenetics for restoration of vision

**Fig. 4:**
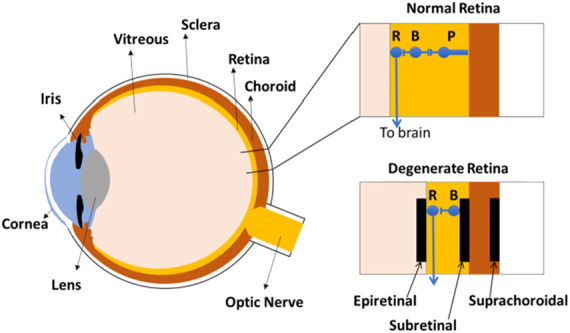
Cross section of the eye showing the relative positions of the retinal ganglion cells (R), bipolar cells (B) and photoreceptors (P) in a normal retina. In a degenerated retina, electrode arrays could be placed in epiretinal, subretinal or suprachoroidal positions

Vision loss can arise from the loss of photoreceptors in degenerative retinal diseases such as macular degeneration or retinitis pigmentosa. However, the inner retinal neurons and retinal ganglion cells often remain intact and are the target of electrical devices [74]. Electrical retinal arrays of up to 1500 electrodes have been implanted into people with damaged photoreceptors for direct stimulation of retinal bipolar cells or retinal ganglion cells [75]. The retinal neural prosthesis may be placed on top of the retina (epiretinal), under the inner nuclear layer (subretinal) or between the choroid and sclera (suprachoroidal) (Fig. 4). Epiretinal arrays target the retinal ganglion cells and are relatively easy to implant or remove, but passing axons may also be unintentionally stimulated resulting in a distorted visual response. Nonetheless, patients have performed relatively well in object discrimination tasks [76]. Subretinal implants offer greater stability and fixation in the retina but are more limited in size and more difficult to implant or remove if required. Recipients of electrical subretinal arrays have demonstrated the ability to see and recognise large format letters [75]. The suprachoroidal implantation location is safer but has a larger distance between the device and the target neurons which increases the stimulation thresholds, but visual percepts have been reported from implanted recipients [77–79].

While electrical retinal prostheses have restored basic, low-resolution visual percepts to the recipients [75, 80–82], the technology is far from restoring vision for safe navigation in natural environments or reading and are still at the proof-of-concept stage. Increased precision of neural stimulation in the retina may enable the use of more independent channels and help to minimise side-effects. Optical stimulation of retinal tissue is one potential solution that is under investigation. In the retina, channelrhodopsin, or even rod opsins normally found in human retinal rod cells, may be targeted to the ON-bipolar cells, which are still present in late stages of disease, with restoration of ON and OFF responses and light-mediated behavioural responses in blind mice [83–85]. Retinal ganglion cells may also be transfected with long-term expression, but with very high thresholds of activation [86].

Channelrhodopsins typically have a low light sensitivity due to the lack of a cascade amplification system, therefore an external device is likely to be required to activate the cells. However, the amount of light required for activation in the retina may be phototoxic to the cells [87]. Red shifted opsins can help reduce phototoxicity and have been shown to restore light responses in multiple models [88]. Issues of scattering and absorption issues are also reduced with red shifted opsins. Recent efforts have attempted to obtain functional responses at lower light levels. One such study has used a Ca^2+^-permeable channelrhodopsin (CatCh) that is 70 times more sensitive than channelrhodopsin, generating responses in monkey retinal ganglion cells with light intensities below illumination safety limits [87].

There are currently clinical trials (detailed in Section 6) examining viral-vector mediated channelrhodopsin expression in the retina for retinitis pigmentosa, which will establish safety and efficacy of optogenetic-based therapies for vision restoration that will be of interest for the fields of optogenetics and optical neural stimulation in general.

### 4.3 Deep brain stimulation

Electrical stimulation of neurons in the subthalamic nucleus alleviates tremors, stiffness and postural instability symptoms in people with movement disorders and neurological conditions such as Parkinson's disease. However, the presence of nearby major neural fiber pathways can confound the area of activation and create a trade-off between maximum therapeutic effect and minimal side-effects [89, 90]. An optogenetic approach was used to understand the mechanism of DBS and identify the target cell types. When the inhibitory halorhodopsin was expressed in excitatory glutaminergic neurons of the subthalamic nucleus, light-mediated inhibition via an implanted light source was found to have no effect on the induced Parkinsonian symptoms in rats [91]. Likewise, expression of the excitatory ChR2 had no effect when stimulated by high frequency light pulses. Conversely, optical stimulation of ChR2-expressing axonal efferents that projected into the subthalamic nucleus reversed the induced Parkinsonian symptoms, as did optical stimulation of the ChR2 positive cell bodies in layer V of the primary motor cortex, demonstrating that the targets of DBS may not exclusively originate in the subthalamic nucleus [91]. There is strong interest in the development of non-invasive mechanisms of light delivery to the brain for DBS, which is hampered by the strong scattering and absorption of blue-green wavelengths and the brain's high lipid content. Making use of the greater tissue penetration of near infrared light (NIR), lanthanide-doped up-conversion nanoparticles can be used to convert NIR to visible light which in turn can activate channelrhodopsin ion channels [92]. Applying this technology in mice, the ventral tegmental area of the brain was locally injected with an AAV encoding ChR2 and lanthanide-doped silica-coated nanoparticles. NIR pulses applied trans-cranially were subsequently up-converted to blue light with sufficient intensity to activate ChR2 in transfected neurons. Optogenetic inhibition was also demonstrated by substituting the ChR2 for an archaerhodopsin and using green-emitting up-conversion nanoparticles [93]. While the technique has a long way to go before potential clinical applications, it demonstrates that non-invasive solutions could be developed for delivery of optical stimulation to deep layer tissues, but invasive delivery of the viral vectors and nanoparticles are still required.

Currently, there is increasing research on optogenetics for deep brain stimulation applications such as Parkinson's disease and epilepsy with an emphasis on the potential for selective activation or inhibition and for closed loop control, whereby electrical signals from the brain can be recorded without the interference that normally occurs from stimulating with electrical pulses. It is apparent that further research on the fundamental mechanisms of deep brain stimulation is still required to implement optogenetic therapies to these systems, such as knowledge of the neuronal subtypes involved and even whether excitation or inhibition is required to control the symptoms, but optogenetics can also provide the tools to untangle these issues [94].

## 5 Optical interface development and considerations

**Fig. 5:**
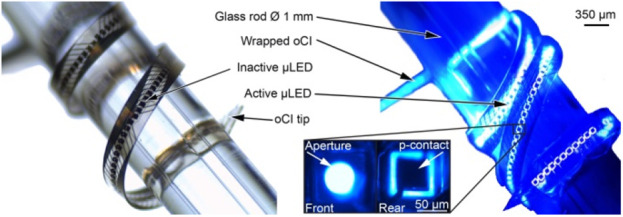
High density micro LEDs mounted on a flexible substrate shown wrapped around a 1 mm glass rod under normal light conditions and with the micro LEDs emitting 462 nm light. Image originally published by Klein et al. [96] in Frontiers in Neuroscience and reproduced here with permission

If precise neural stimulation leads to a greater number of channels providing independent information, there must be simultaneous development of optical arrays with a high channel count. The cochlea is particularly challenging with its spiraling anatomy and delicate internal structures dictating flexible materials. Glass optical fibers used experimentally are precluded from clinical use due to their lack of flexibility and the need for an external light source. An alternative solution, light-emitting diodes (LEDs), presents challenges in terms of dimensions, flexibility and safe encapsulation to protect the body and the electronic components. An optical cochlear implant with 15 thin-film gallium nitride LEDs (50 × 50 μm^2^) on a polyimide substrate was initially developed using wafer-level packaging technique and tested in a mouse model [95]. However, the array suffered from thermomechanical issues which were later addressed by replacing the polyimide with a transparent epoxy material resulting in an array of 144 individually controlled micro LEDs with 50 μm diameter apertures. The array was flexible enough to wind around a 1 mm diameter glass rod, simulating the spiral of a cochlea, and could deliver enough optical power for neural stimulation in mice and gerbils [96] (Fig. 5). The field is continuing to develop and test materials that can safely and effectively deliver sufficient light to neural tissue, which will then require clinical trials to address safety and efficacy in humans. Ultimately, optical devices would need to match the robustness and safety of their electrical counterparts which are required to reliably stimulate neurons over the life span of a recipient.

Light propagation through tissue is influenced by scattering and absorption [97], both of which affect the amount of light that reaches the target. Longer wavelengths such as red light penetrate further and scatter less. Absorption is dominated by water for infrared light and by haemaglobin for visible light. Light with 800–1000 nm wavelength is ideal for high penetration and low scattering. In the cochlea, light would need to penetrate the perilymphatic fluid and a thin 6–25 μm bone layer [98] before reaching the auditory neurons. In the retina, light would need to penetrate no more than ∼250 μm if delivered from an epiretinal device. However, as the body attempts to isolate itself from implanted devices, a fibrotic tissue reaction often encapsulates the device. For the cochlear implant, the fibrous capsule sometimes fills the entire perilymphatic space and is sometimes accompanied by bone formation, both of which can lead to higher electrical thresholds over time and has been associated with the loss of residual hearing in the implanted ear [99]. For a chronically implanted optical array, a fibrous tissue capsule is predicted to influence the amount of light required to activate target neurons. Pharmacological solutions such as treatment with steroids have been shown to reduce the foreign body response in human cochlear implant recipients [100] and therefore may provide some utility in minimising the impact of tissue fibrosis on the efficacy of optical stimulation.

Thermal load from micro-LED stimulation in confined tissues such as the cochlea is of equal concern for the optogenetic stimulation as it is for INS, as is phototoxicity from the use of high intensity visible light that is well known in the field of live cell microscopy [101, 102]. Phototoxicity can be reduced through the use of red-shifted opsins [88] and the presentation of fewer optical pulses at lower power levels, for example, by combining optical and electrical stimulation [103], in order to exploit the speed and efficiency of electrical stimulation and the precision of optical stimulation. In cultured auditory neurons expressing the H134R variant of ChR2, subthreshold pulses of optical stimuli raised the excitability of neurons such that subthreshold electrical stimuli (e.g. 40% of threshold) activated the neurons [103], an effect that has been reported for the peripheral nervous system when INS was combined with electrical stimulation [19, 104]. Furthermore, the rate following ability of auditory neurons was three-fold higher when combining sub-threshold electrical and optical inputs, but the impact on spread of activation remains to be tested in vivo.

One of the problems with devices designed to restore vision is that many patients retain some useful visual percepts. For example, patients with age-related macular degeneration lose their central vision but retain peripheral vision, which remains very helpful for navigation. Devices that require the use of light to stimulate devices or opsins need to take into account the risk of overwhelming the residual light responses still present in the visually impaired eye. For all implanted devices such as retinal prostheses or cochlear implants, the surgery needs to avoid damage to residual function. For optogenetics, there is the additional consideration of the safety issues associated with the viral vectors used and the potential toxicity of activation strategies (high light levels) and the opsins themselves.

## 6 Clinical and commercial development

Progress in pre-clinical animal research models has spurred the move towards human clinical trials of optical-based neural stimulation for the treatment of neurological impairment, primarily in the retina.

Multiple companies are developing optogenetic solutions for vision restoration. Retrosense therapeutics, acquired by Allergan in 2017, sponsored a clinical trial in which the ChR2 gene was introduced into the vitreous of the eye of patients with advanced retinitis pigmentosa via an AAV. The phase I/IIa trial is examining the safety and tolerability of the viral gene therapy. Although expression of the ChR2 gene in retinal ganglion cells will render the cells responsive to light, a device will be required to capture and amplify the visual information to a level that would activate the ion channels. If successful, the trial will be extended to include dry age-related macular degeneration. Similarly, GenSight Biologics is offering an optogenetics solution for retinal degenerative diseases such as retinitis pigmentosa based on ChrimsonR. After showing that ChrimsonR could restore light sensitivity in blind mice [83], an intravitreal AAV2.7m8 gene therapy (CAG promoter) is being trialed in macaques with resulting ChrimsonR expression in retinal ganglion cells. The retinal ganglion cells were shown to be responsive to light and the injection was well tolerated [105]. In newer developments, opsins with improved light sensitivity have been shown to restore vision (in electrophysiological and behavioural studies) in mice with ambient light, eliminating the need for an associated device. Large photocurrent variants of ChR2 (CoChR) were produced with slower off-kinetics, therefore making them more sensitive to light [106], capitalising on the fact that the visual system can tolerate slower kinetics compared to the auditory system. Using a different approach, Acucela is targeting a different retinal cell type, the ON bipolar cells, with the premise that there will be better signal processing and amplification and no need for an external light device [85, 107].

## 7 Conclusion

The potential applications of electrical stimulation are expanding as concepts from the cochlear implant are applied to more and more clinical indications. This only increases the need for greater precision and specificity of neural activation to drive improvements in patient outcomes. Optical stimulation presents a promising alternative and paradigm-changing approach. The evidence of greater precision of activation is currently off-set by the need to permanently modify the neurons (in the case of optogenetics) and a higher overall power requirement. Continued advances in this fast-paced field and positive outcomes from clinical trials will progress towards the translation of optical neural stimulation for a wide range of neurological conditions.
